# Urinary creatinine in very low gestational age neonates: importance for interpretation of creatinine-normalized urinary amino acids

**DOI:** 10.1016/j.jped.2026.101600

**Published:** 2026-09-07

**Authors:** Jolanta Bugajska, Joanna Berska, Magdalena Zasada, Przemko Kwinta, Przemysław Tomasik, Krystyna Sztefko

**Affiliations:** a Jagiellonian University Medical College, Institute of Pediatrics, Department of Clinical Biochemistry, Krakow, Poland; b University Children's Hospital, Department of Clinical Biochemistry, Krakow, Poland; c Poland Jagiellonian University, Jagiellonian University Medical College, Institute of Pediatrics, Department of Pediatrics, Krakow, Poland

**Keywords:** Very low gestational age neonates, Urinary creatinine, Urinary amino acids, Biological variation, Gestational age

## Abstract

**Objectives:**

Urinary creatinine concentration is widely used to normalize urinary analytes. However, its reliability in preterm neonates remains uncertain. This study aimed to evaluate urinary creatinine concentrations in very low gestational age (VLGA) neonates and assess its impact on the interpretation of urinary creatinine normalized urinary amino acids.

**Methods:**

Spot urine samples were collected from VLGA neonates (22–31 weeks gestational age, n = 32) and full-term neonates (≥ 37 weeks, n = 22) during the first days of life. Urinary creatinine was measured serially, and biological variation (within- and between-subject) was assessed. Urinary amino acids were measured on the 4th day in full-term neonates and in VLGA neonates at term-equivalent age using LC-MS/MS and expressed both as absolute concentrations (µmol/l) and normalized to creatinine (mmol/mol creatinine).

**Results:**

Urinary creatinine concentrations were significantly lower in VLGA neonates compared to full-term neonates during the first days of life (p ≤ 0.004). A positive correlation between birth weight and urinary creatinine was observed (r = 0.46, p = 0.002). Within-subject biological variation was lower in VLGA neonates, while between-subject variation was comparable between groups. Urinary amino acid concentrations expressed in μmol/l did not differ significantly between groups, however, when expressed relative to creatinine, multiple significant differences emerged.

**Conclusions:**

Gestational age should be considered when interpreting creatinine normalized urinary amino acids concentrations in neonates.

## Introduction

Creatinine is a small molecule cleared from plasma almost exclusively by glomerular filtration with minimal tubular secretion. It is widely used in newborns and infants to normalize the excretion of urinary analytes, including catecholamines in neuroblastoma diagnosis [1], urinary amino acids [2], and proteinuria or albuminuria in suspected glomerular disease or injury [3,4].

Amino acid concentrations are most commonly measured in fasting plasma, however frequent blood sampling in newborns may cause significant blood loss and iatrogenic anaemia. Moreover, some metabolic disorders can only be diagnosed by measuring specific urinary amino acids, such as cystinuria [5], lysinuric protein intolerance [6], hyperornithinemia-hyperammonemia-homocitrullinuria (HHH) syndrome [7], and alkaptonuria [8]. Urinary amino acid analysis is also useful for assessing renal tubular function, including proximal tubular dysfunction (e.g., Fanconi syndrome) [9] kidney maturation [10] and amino acid imbalance related to neurodevelopmental disorders [11].

Interpretation of urinary amino acid profiles is challenging. Both spot and 24-hour urine samples are used, with only minor differences reported [12,13]. As creatinine is commonly used for normalization, understanding its variability in neonates is essential. Data in preterm infants are limited.

This study aimed to: (1) assess trends in urinary creatinine in very low gestational age (VLGA) and full-term neonates during the first days of life, and (2) evaluate the role of creatinine in normalizing urinary amino acid concentrations.

## Materials and methods

Spot urine samples were collected from the neonates treated at the Neonatal Intensive Care Unit (NICU) at the Institute of Paediatrics of Jagiellonian University Medical College in Krakow, Poland. Urine was collected non-invasively with a sterile cotton ball (Paul Hartmann, Pabianice, Poland) or via sterilized urine bag (Zarys, Zabrze, Poland). Any urine sample contaminated with stool was discarded, and a new sample was collected.

Two cohorts were enrolled (April 2021–July 2023): VLGA neonates (a gestational age (GA) 22–31 weeks, n = 32) and full-term neonates (GA ≥ 37 weeks, n = 22). Infants with congenital anomalies or suspected metabolic/genetic disorders were excluded.

Urine for creatinine analysis was collected on 1st, 2nd, 3rd, 4th, 6th, and 8th day of life; additionally on day 28th day of life and at term-equivalent age (TEA) in VLGA infants. Due to the lack of possibility to get good sample quality, few urine spots were missing. From 19 VLGA infants and from 8 full-term infants’ urine was collected at each planned point in time. Creatinine was measured using a dry chemistry analyzer (Vitros 4600, Ortho Clinical Diagnostics Inc., Rochester, NY, USA).

Urinary amino acids were measured in 11 VLGA infants at term-equivalent age (TEA), as at this time they were not receiving parenteral nutrition. In six healthy full-term neonates, urinary amino acid concentrations were measured on the fourth day of life. The fourth day was selected to minimize the potential influence of maternal creatinine on urinary amino acid concentrations expressed relative to creatinine levels. After collection, urine samples for amino acid analysis were centrifuged at 2600 × g for 10 min at 4 °C and stored at −80 °C until analysis. Urine amino acids concentrations were measured using a highly selective liquid chromatography-tandem mass spectrometry method (LC-MS/MS, 1260 Infinity II, 6460 QTRAP; Agilent Technologies, Waldbronn, Germany) with a quantitative amino acid analysis kit (Jasem, Istanbul, Turkey). The following amino acids were measured: tryptophan, taurine, phenylalanine, threonine, leucine, isoleucine, methionine, valine, ornithine, γ-aminobutyric acid, glutamic acid, aspartic acid, 2-aminobutyric acid, tyrosine, serine, alanine, glycine, asparagine, glutamine, proline, homocitrulline, citrulline, cystine, cystathionine, arginine, histidine, lysine, 1-methylhistidine, 3-methylhistidine, and anserine. Method for amino acids analysis used in the present study is under control of the European Research Network for Inherited Disorders of Metabolism (ERNDIM, MCA Lab, Netherlands). The study was conducted according to the guidelines of the Declaration of Helsinki and approved by the Jagiellonian University Ethics Committee (Jagiellonian University Bioethical Committee, Krakow, Poland, approval No 1072.6120.336.2020; No 1072.6120.95.2023), written informed consent was obtained from the infants’ parents.

### Statistical analysis

Data were analyzed using Statistica 13 (StatSoft, Krakow, Poland) and Microsoft Excel 2010. Biological variation (CV_I_, CV_G_) and the index of individuality (II) were calculated. Data are presented as median (interquartile range, IQR). Spearman’s correlation was used to assess the relationship between birth weight and urinary creatinine. The Shapiro–Wilk test was used to evaluate data distribution. Variables with an approximately normal distribution (tryptophan, taurine, phenylalanine, threonine, γ-aminobutyric acid, aspartic acid, tyrosine, serine, alanine, glycine, asparagine, glutamine, proline, cystine, histidine, 3-methylhistidine, anserine, and total amino acids) were compared between groups using *t*-test. Variables that did not meet the assumption of normality (leucine, isoleucine, methionine, valine, ornithine, glutamic acid, 2-aminobutyric acid, homocitrulline, citrulline, cystathionine, arginine, lysine, 1-methylhistidine, and creatinine) were compared using the Mann–Whitney U test. To account for multiple comparisons, P values were adjusted using the Benjamini–Hochberg false discovery rate (FDR) procedure. Statistical significance was set at p < 0.05.

## Results

On the first, second, third, fourth and eighth day of life urinary creatinine concentration in full-term neonates was significantly higher compared with VLGA neonates (p = 0.004 – p ≤ 0.001) (Table 1).

**Table 1:** The median (interquartile range) values of urinary concentration of creatinine (mmol/l) in very low gestational age (VLGA) neonates on the 1st, 2nd, 3rd, 4th, 6th, 8th, 28th day of life and term-equivalent age (TEA) and in full-term neonates on the 1st, 2nd, 3rd, 4th, 6th, 8th day of life.

day of life	very low gestational age neonates		full-term neonates		p
	n	Creatinine Median (Interquartile range) [mmol/l]	n	Creatinine Median (Interquartile range) [mmol/l]	
1	28	0.849 (0.476 - 1.275)	14	1.831 (1.090 – 2.195)	<0.001
2	31	0.803 (0.640 −1.265)	21	1.736 (1.350 – 2.902)	<0.001
3	32	0.849 (0.710 −1.335)	19	1.509 (0.926 – 2.245)	0.004
4	31	0.913 (0.582 −1.312)	21	1.271 (1.113 – 2.244)	0.001
6	31	0.794 (0.614 −1.085)	15	0.786 (0.552 – 1.005)	0.468
8	32	0.714 (0.612 −0.862)	13	1.200 (0.885 – 1.419)	0.001
28	30	0.735 (0.468 −1.155)		NA	
TEA	26	0.834 (0.450 −1.304)		NA	

n – number of urine samples (not all samples were available due to insufficient sample quality).

Within subject biological variation for spot urine creatinine concentration, using data obtained from all newborns, was significantly lower in VLGA neonates as compared to full term neonates (p < 0.004). Between subject variation was similar in both groups. Also, index of individuality, calculated as the ratio of within subject variability to between subject variability was similar in both groups (Table 2).

**Table 2:** Within-subject (CV _I_) and between-subject (CV_G_) biological variations, index of individuality (II) of creatinine in spot urine creatinine samples from very low gestational age (VLGA) and full-term newborns.

	Number of subjects	CV _I_ (95% CI), %	CV_G_ (95% CI), %	Index of individuality (II)
VLGA neonates	32	35.1 (30.3 - 40.0) *	48.2 (38.0 −58.3)	0.73 (0.63–0.83)
Full-term neonates	22	48.6 (39.7 - 57.5)	48.0 (37.8 −58.3)	1.00 (0.82–1.18)

⁎ - significant difference between VLGA neonates and full-term neonates. CV _I_ – within-subject biological variations. CV_G_ - between-subject biological variations. CI – confidence interval.

**Figure 1:**
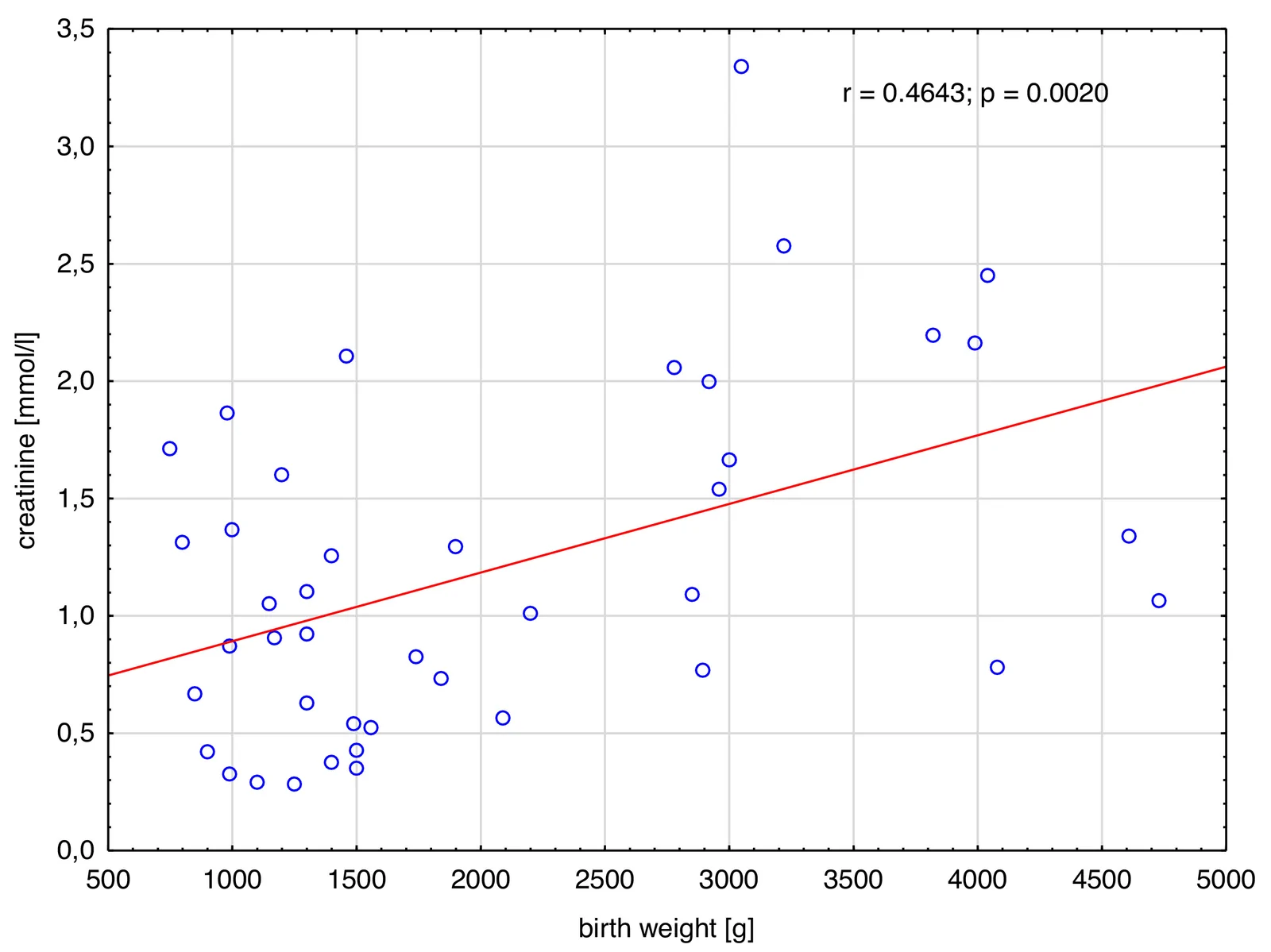
Spearman's correlation between birth weight and urinary creatinine concentration on the 1st day of life (all newborns).

The mean birth weight of the infants was 1369±380 g (VLGA) and 3415±595 g (full-term). For all newborns a positive correlation between birth weight and urinary creatinine on the 1st day of life (r = 0.46, p = 0.002) (Figure 1) was observed.

**Figure 2:**
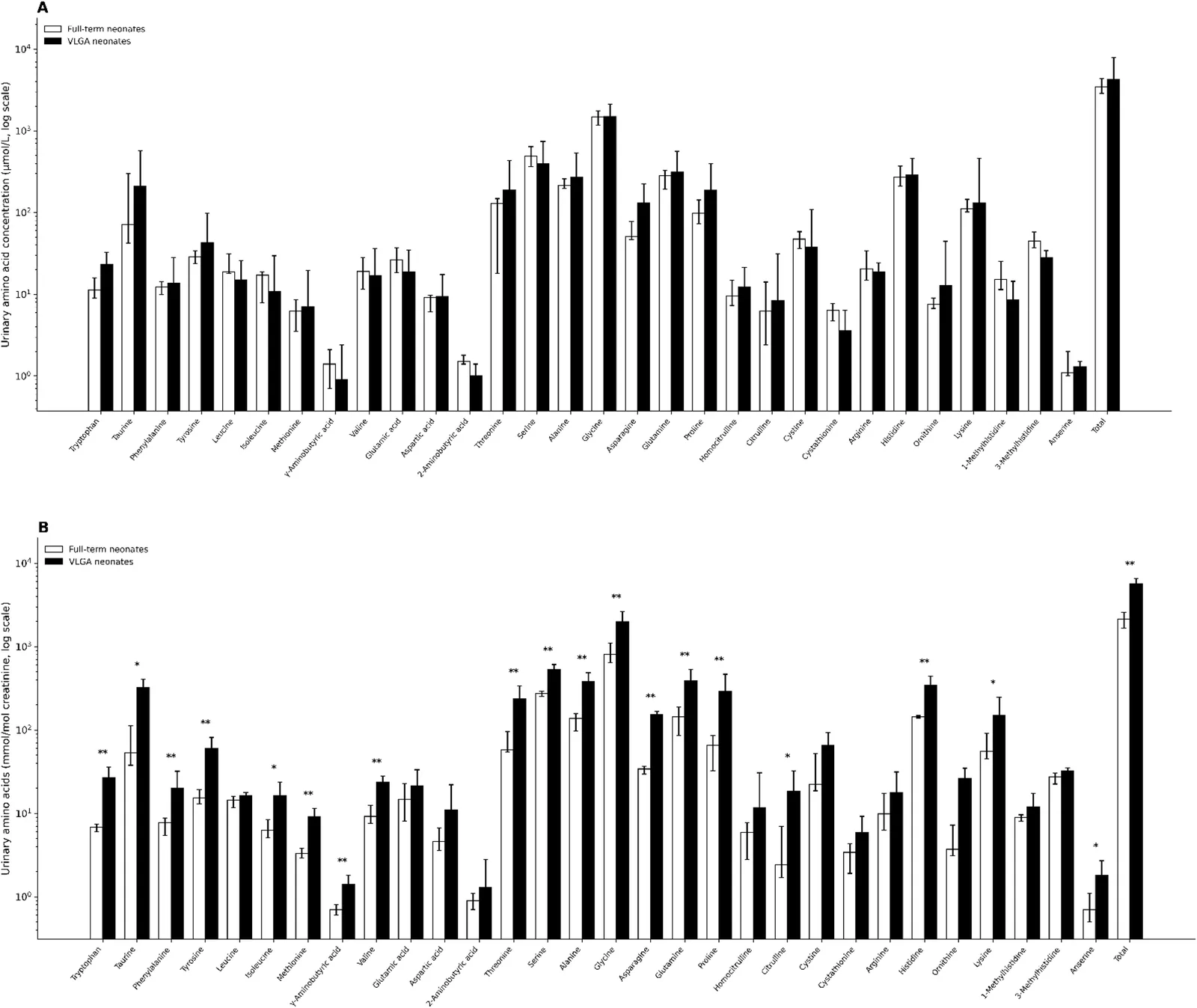
Urinary amino acids on the fourth day of life in full-term neonates and at term-equivalent age (TEA) in very-low-gestational-age neonates not receiving parenteral nutrition. A - Urinary amino acids expressed as absolute concentrations (µmol/l). B - Urinary amino acids expressed as creatinine normalized values (mmol/mol creatinine). White and black bars represent the median values. Error bars indicate the interquartile range. Full term neonates and VLGA neonates were compared using the *t*-test or the Mann-Whitney U test, depending on data distribution. P values were adjusted for multiple comparisons using the Benjamini-Hochberg false discovery rate procedure. *q < 0.05, **q < 0.01.

Urinary amino acids concentrations (µmol/l) were comparable between groups (Figure 2A). However, after normalization to creatinine, significant differences were observed for tryptophan, taurine, phenylalanine, tyrosine, isoleucine, methionine, γ-aminobutyric acid, valine, threonine, serine, alanine, glycine, asparagine, glutamine, proline, citrulline, histidine, lysine, and anserine (q < 0.01 to q < 0.05) (Figure 2B).

## Discussion

High neonatal creatinine levels reflect maternal transfer [14]. Allegaert et al. [15] noticed that the peak of serum creatinine value occurred on the 3rd day of life in ≤ 32-week neonates and on the 2nd day of life in full-term neonates. Subsequently, serum creatinine concentrations decreased and stabilized around the 5th–6th day of life [15]. Ponthier et al. [3] observed lower urinary creatinine excretion in newborns with a gestational age (GA) <29 weeks and in those with a GA between 29 and 33 weeks, both during the first 48 h after birth and between 72 and 120 h after birth, compared to newborns with a GA ≥ 33 weeks. Our findings confirm the lower urinary concentration in VLGA neonates compared to full-term neonates on the 1st, 2nd, 3rd, 4th and 8th days of life. The observation is linked to a positive correlation between urinary creatinine and newborn body weight. Based on research conducted in 48 healthy full-term neonates aged 1 to 6 days (median 2.4 days) and in 168 healthy children with a median age of 1.5 years (from one month to 3 years) Matos et al. [16] do not recommend using urinary creatinine to standardize urinary solute excretion of in the first week of life, because of high and variable urinary creatinine concentration during the first days of life. In another words, urinary creatinine concentration is not reliable reference value for standardizing urinary solute excretion in spot urine samples during the first week of life.

In VLGA infants after 28 days and at TEA urine creatinine remains low. It is very important from a diagnostic point of view when other urinary markers are measured and expressed relatively to creatinine and “normal” values as reference point are taken. The laboratory should ask for clinical information regarding prematurity of the child.

Biological variation estimates derived from meta-analyses conducted in accordance with the BIVAC (Biological Variation Data Critical Appraisal Checklist) criteria show that, in adults, creatinine measured in random urine samples exhibits CV_I_ (%, 95%CI) of 41.8 (36.3–47.4) and CV_G_ (%, 95%CI) of 42.1 (35.6–56.8) [17]. We obtained comparable results. Urinary creatinine concentration is strongly influenced by hydration status, physical activity, and diet [18,19]. In neonates, lifestyle-related determinants of urinary creatinine variability are relatively limited due to their physiological uniformity; however, variability is influenced by renal immaturity and rapid developmental changes.

The index of individuality is a well-established tool in clinical diagnostics that indicates the usefulness of population-based reference intervals for interpreting results of a given analyte. It is calculated as the ratio of within-subject (intra-individual) variation to between-subject variation. A high II (> 1.4) suggests that population-based reference intervals are appropriate for laboratory data interpretation, whereas a low II (< 0.6) indicates their limited usefulness; in such cases, personalized reference intervals are recommended [20,21]. Yılmaz Çalık et al. [22] reported an II for spot urine creatinine of 0.59 in adults. Although the observed index of individuality (0.73 in VLGA neonates and 1.00 in full-term neonates) do not unequivocally support replacing population-based reference intervals, they suggest that individualized interpretation of serial measurements may provide greater clinical value, particularly during the longitudinal follow-up of VLGA infants, in whom renal function and creatinine excretion change rapidly after birth. In this setting, longitudinal evaluation of serial measurements may provide additional clinical value, especially when urinary creatinine is used to normalize other urinary analytes, including amino acids, tubular injury biomarkers (e.g., NGAL and KIM-1), and urine protein-to-creatinine or albumin-to-creatinine ratios commonly used in neonatal nephrology [[23], [24], [25]]. It may also improve interpretation of serial measurements in infants receiving potentially nephrotoxic medications, those monitored for acute kidney injury, or those undergoing evaluation for renal tubular dysfunction or inherited metabolic disorders, in whom urinary amino acid profiles are assessed repeatedly [26,27]. In these clinical situations, comparison with an individual's previous results, or the use of reference change values where available, may provide more clinically meaningful information than reliance solely on population-based reference intervals [26,28].

The interpretation of urinary analytes expressed relative to creatinine is complicated by low urinary creatinine concentrations observed in VLGA neonates, which may remain low even beyond the first month of life. This limitation challenges the interpretation of amino acid concentrations during the first months of life in neonates. A lack of age- and gestational age specific reference ranges, particularly for preterm infants, limits the diagnostic utility of measured values. Parvy et al. [12] excluded premature infants from their study group when developing reference values for free amino acids in first morning urine specimens. As a result, the range of reference values for urine amino acids normalized to urinary creatinine obtained by Parvy et al. [12] for children under one month of age has narrower range compared with those reported by Venta et al [13]. who did not exclude premature infants.

We demonstrated that VLGA neonates have lower urinary creatinine concentrations at term-equivalent age than full-term infants on the 4th day of life, while urinary amino acid levels expressed in µmol/l remain comparable between these groups. This indicates that the apparent differences in amino acid excretion observed after normalization to creatinine are largely driven by reduced creatinine excretion rather than true metabolic alterations. This again highlights that prematurity is one of the important ‘preanalytical’ factor which should be taken into consideration when interpreting the urinary markers expressed relative to creatinine.

From a clinical perspective, this finding is particularly relevant, as it suggests that creatinine normalized urinary amino acid values may overestimate amino acids excretion in VLGA neonates and consequently may lead to misinterpretation of metabolic status or unnecessary diagnostic concern.

It is indicated that in neonates the results of urinary amino acids should not be interpreted using a single normalization strategy in all clinical settings. Absolute concentrations of urinary amino acids (µmol/l) may be preferable when urinary creatinine excretion is low, rapidly changing, or strongly influenced by developmental factors, as is the case in very preterm infants during the early postnatal period or when comparing neonates of substantially different gestational ages. Until gestational age and postnatal age specific reference intervals become available, we recommend reporting both absolute and creatinine-normalized amino acid concentrations, together with gestational age, postnatal age, and relevant clinical information. Urinary amino acids results expressed as creatinine normalized values should be interpreted with caution, and should not be used as the sole measure, in infants with rapidly changing renal function, very low urinary creatinine concentrations, substantial differences in muscle mass.

Reliable urine normalization methods are crucial in metabolomics, particularly when analyses rely on spot urine samples. Osmolality is commonly used to account for urine dilution and may be more appropriate than creatinine in populations with highly variable creatinine excretion, such as neonates. In healthy adults, osmolality-based normalization has been shown to improve the performance of untargeted urine metabolomics and demonstrated higher analytical precision than creatinine, while being less affected by age, muscle mass, and dietary intake [29,30]. However, measuring osmolality requires dedicated equipment. Urine specific gravity has also been used as a simpler surrogate of urine concentration [31], although it is less accurate because it reflects urine density rather than solute concentration and is influenced by the presence of large molecules.

Cook et al. [32] demonstrated that statistical significance may depend on the normalization method applied, emphasizing that no single approach is universally optimal. In adults, 24-hour urine collection is considered the reference method for estimating urinary metabolite excretion; however, it is rarely feasible in neonates, particularly in preterm infants. Consequently, urinary creatinine is commonly used for normalization despite its limitations. During the early neonatal period, urinary creatinine excretion changes rapidly after birth and is influenced by gestational age, muscle mass, renal maturation, and maternal creatinine.

Body size-based normalization (e.g., weight-adjusted excretion) also appears to be unsuitable in very low gestational age infants because urinary creatinine remains low despite increasing body weight. Therefore, gestational age- and postnatal age-specific reference intervals may provide a more physiologically appropriate framework for interpreting urinary metabolite concentrations.

Urinary cystatin C is currently used primarily as a biomarker of kidney function and tubular injury rather than as a normalization factor. Although it appears relatively stable during early postnatal life in preterm infants [33], there is currently no evidence supporting its use for normalization of urinary metabolite concentrations. This possibility warrants further investigation.

Most studies evaluating urine normalization strategies in metabolomics have been performed in adults. Only a few have reported reference values for absolute and creatinine-normalized urinary metabolite concentrations in healthy full-term neonates [34], and, to our knowledge, no such studies have established reference values for preterm neonates.

The marked differences in urinary creatinine excretion between VLGA infants and full-term neonates indicate that caution is warranted when expressing urinary metabolites, including amino acids, relative to creatinine. These findings support the need to establish gestational age-specific reference values and to evaluate alternative normalization methods in preterm populations.

Alternative approaches, such as the use of absolute concentrations or gestational age-specific reference intervals, should therefore be considered when interpreting urinary biomarkers in preterm infants.

This study has several limitations. First, the relatively small sample size, particularly in the subgroups in which urinary amino acids were measured (VLGA, n = 11; full-term neonates, n = 6), limits the statistical power of the analyses and increases the risk of both type I and type II errors. Consequently, the findings should be interpreted with caution and cannot be generalized to the broader population of preterm and full-term neonates. Furthermore, the limited sample size may have affected the precision of the estimated biological variation components and the index of individuality.

Second, urinary amino acid concentrations were compared between full-term neonates on the fourth day of life and VLGA infants at term-equivalent age. Although TEA represents a comparable stage of developmental maturity, the groups differed substantially in postnatal age. Therefore, postnatal adaptation, renal functional maturation, and cumulative extrauterine development may have influenced urinary amino acid excretion independently of gestational maturity. The fourth day of life was deliberately selected for full-term neonates to minimize the influence of maternally derived creatinine on creatinine-normalized urinary amino acid concentrations. Nevertheless, the inability to compare infants at the same postnatal age or corrected age should be considered when interpreting the observed differences.

Another limitation is that urine samples could not be collected at all planned time points, mainly because of insufficient urine volume and occasional stool contamination, which are common challenges in urine collection from very preterm infants. Consequently, the number of observations differed between sampling time points, and statistical comparisons were performed using the available data only, without imputation of missing values. The reduced sample size at some time points further decreased the statistical power of the analyses and may have increased the risk of type II error. Moreover, creatinine measurements were performed as single determinations rather than in duplicate. These factors may have affected the precision of the estimated within-subject (CV_I_) and between-subject (CV_G_) biological variation, as well as the index of individuality (II).

Future studies including larger, preferably multicenter, cohorts, more complete longitudinal sampling, and longitudinal comparisons performed at comparable postnatal and corrected ages are warranted to validate the present findings, establish robust gestational age-specific reference intervals, and better distinguish the effects of gestational maturity from those of postnatal development.

A major strength of this study is the quantitative assessment of dynamic changes in urinary creatinine during the first six days after birth in very low gestational age neonates and term-born neonates. In addition, all urine samples from both newborn groups were collected during the same time period, with minimal preanalytical errors, thereby improving the comparability and homogeneity of the measurements.

## Conclusions

Reliable measurement of urinary creatinine concentrations in newborns and neonates, with special attention to samples taken from very low gestational age neonates, is necessary when urinary solutes are normalized to creatinine. Gestational age should be always considered when interpreting urine amino acids concentrations in neonates suspected of aminoaciduria or other disorders of amino acid metabolism.

## Funding sources

The study was supported by a grant from Jagiellonian University (N41/DBS/001100).

## CRediT authorship contribution statement

**Jolanta Bugajska:** Conceptualization, Methodology, Investigation, Visualization, Funding acquisition, Project administration, Writing – original draft, Writing – review & editing. **Joanna Berska:** Methodology, Investigation, Writing – review & editing. **Magdalena Zasada:** Methodology, Investigation, Writing – review & editing. **Przemko Kwinta:** Methodology, Project administration, Writing – review & editing. **Przemysław Tomasik:** Project administration, Writing – review & editing. **Krystyna Sztefko:** Conceptualization, Supervision, Writing – review & editing.

## Conflicts of interest

The authors declare no conflicts of interest.

## Data Availability

The data that support the findings of this study are available from the corresponding author.
